# Hybrid Laser Cleaning of Carbon Deposits on N52B30 Engine Piston Crowns: Multi-Objective Optimization via Response Surface Methodology

**DOI:** 10.3390/ma18153626

**Published:** 2025-08-01

**Authors:** Yishun Su, Liang Wang, Zhehe Yao, Qunli Zhang, Zhijun Chen, Jiawei Duan, Tingqing Ye, Jianhua Yao

**Affiliations:** 1 College of Mechanical Engineering, Zhejiang University of Technology, Hangzhou 310023, China; yissu@zjvtit.edu.cn (Y.S.); laserwang@zjut.edu.cn (L.W.); zhyao@zjut.edu.cn (Z.Y.); zql@zjut.edu.cn (Q.Z.); roll@zjut.edu.cn (Z.C.); 2 Automobile School, Zhejiang Institute of Communications, Hangzhou 311112, China; 3 Institute of Laser Advanced Manufacturing, Zhejiang University of Technology, Hangzhou 310023, China; 4 DITT Hangzhou Digital Technology Co., Ltd., Hangzhou 310020, China; duanjw@zstcloud.com; 5 Hangzhou CRRC Vehicle Co., Ltd., Hangzhou 311223, China; altmanye@163.com

**Keywords:** hybrid laser cleaning, engine piston crowns, response surface methodology, surface roughness, carbon residue rate, multi-objective optimization

## Abstract

Carbon deposits on the crown of engine pistons can markedly reduce combustion efficiency and shorten service life. Conventional cleaning techniques often fail to simultaneously ensure a high carbon removal efficiency and maintain optimal surface integrity. To enable efficient and precise carbon removal, this study proposes the application of hybrid laser cleaning—combining continuous-wave (CW) and pulsed lasers—to piston carbon deposit removal, and employs response surface methodology (RSM) for multi-objective process optimization. Using the N52B30 engine piston as the experimental substrate, this study systematically investigates the combined effects of key process parameters—including CW laser power, pulsed laser power, cleaning speed, and pulse repetition frequency—on surface roughness (Sa) and carbon residue rate (RC). Plackett–Burman design was employed to identify significant factors, the method of the steepest ascent was utilized to approximate the optimal region, and a quadratic regression model was constructed using Box–Behnken response surface methodology. The results reveal that the Y-direction cleaning speed and pulsed laser power exert the most pronounced influence on surface roughness (F-values of 112.58 and 34.85, respectively), whereas CW laser power has the strongest effect on the carbon residue rate (F-value of 57.74). The optimized process parameters are as follows: CW laser power set at 625.8 W, pulsed laser power at 250.08 W, Y-direction cleaning speed of 15.00 mm/s, and pulse repetition frequency of 31.54 kHz. Under these conditions, the surface roughness (Sa) is reduced to 0.947 μm, and the carbon residue rate (RC) is lowered to 3.67%, thereby satisfying the service performance requirements for engine pistons. This study offers technical insights into the precise control of the hybrid laser cleaning process and its practical application in engine maintenance and the remanufacturing of end-of-life components.

## 1. Introduction

During engine operation, carbon deposits inevitably accumulate on the piston crown. These deposits degrade combustion efficiency, elevate emissions, and can induce engine knocking, thereby compromising overall engine performance and shortening service life. Traditional cleaning methods—such as chemical solvent cleaning, mechanical grinding, ultrasonic cleaning, and dry ice blasting [1,2]—exhibit inherent limitations. Chemical residues pose environmental hazards [3], mechanical treatments can induce surface damage [4], and none of these techniques enables localized precision cleaning. In recent years, laser cleaning has emerged as a highly reliable and effective solution, owing to its non-abrasive, non-contact nature, minimal thermal impact, and broad material adaptability. At present, laser cleaning technology has been extensively applied for the removal of coatings and oxide layers; however, research on its application for carbon deposit removal remains relatively limited. Y.C. Guan et al. [5] employed pulsed laser cleaning to remove carbon deposits from diesel engine components. Their analysis demonstrated that laser cleaning effectively eliminated Fe_3_C, substantially reduced the concentrations of oxygen-containing functional groups and carboxyl groups, and promoted the formation of Fe_2_O_3_ in the cleaned regions, replacing Fe_3_O_4_. Wang et al. [6] performed laser cleaning experiments targeting carbon deposits and oxides on the surface of GH3030 nickel-based high-temperature alloy. Their results indicated that an appropriate laser spot overlap ratio can effectively remove both carbon deposits and oxides without inducing secondary oxidation of the substrate. Y.L. Qiao et al. [7] investigated the morphological evolution, elemental composition, and relative carbon content of titanium alloy surfaces following pulsed laser cleaning at varying cleaning speeds. The results revealed that at a cleaning speed of 3 cm^2^/s, pronounced laser spot marks and burning phenomena were observed on the surface. In contrast, at a cleaning speed of 7 cm^2^/s, the relative carbon (C) content was significantly reduced, indicating the optimal cleaning performance. From the above research, it can be concluded that most existing studies have focused on using a single-mode laser (such as pulsed or CW lasers) for carbon deposit removal. In many cases, the cleaning parameters are empirically determined, leading to challenges such as uneven cleaning, limited efficiency, and poor control of the heat-affected zone.

It is noteworthy that no studies to date have explored the application of hybrid laser cleaning—integrating CW and pulsed lasers—for carbon deposit removal. This technique leverages the preheating effect of CW lasers to weaken the interfacial bonding strength of carbon deposits, thereby enhancing the ablation and removal efficacy of pulsed lasers. As a result, hybrid laser cleaning improves overall cleaning efficiency, minimizes substrate damage, and is particularly well-suited for addressing complex, multilayer carbon deposits on piston surfaces [8,9]. As early as 1975, Fox et al. [10] pioneered the combination of Q-switched pulsed lasers with CW lasers to enhance the material removal efficiency of steel targets. Subsequently, Pouli et al. [11] employed nanosecond KrF and femtosecond XeCl excimer lasers to remove polymer layers from stone surfaces, and they demonstrated that the pulsed laser ablation effect substantially reduced the interfacial adhesion between the coating and substrate, enabling complete coating delamination. Chen et al. [12] effectively removed paint films from carbon steel surfaces using the synergistic action of CO_2_ and Nd:YAG lasers, and they demonstrated that combining high- and low-power lasers enhances both peeling and ablation mechanisms. Since 2015, Pan et al. [13,14,15], Yuan et al. [16], and Li et al. [17,18,19,20] have investigated the interaction mechanisms of millisecond–nanosecond hybrid lasers with transparent materials, aluminum alloys, and single-crystal silicon. Their studies systematically analyzed the distribution of thermal stress fields, optical absorption characteristics, and microstructural evolution induced by laser irradiation. Pozo-Antonio et al. [21] and Pouli et al. [22] conducted studies employing dual-wavelength lasers to remove blue–black graffiti and coatings from stone surfaces, respectively. Their results demonstrated that hybrid dual-wavelength laser irradiation achieved a superior cleaning performance compared to single-wavelength lasers. In the field of continuous-nanosecond hybrid laser cleaning, Zhang et al. [23] employed this technique to remove paint films from the surface of 6005A aluminum alloy. At a nanosecond laser delay time of 0.4 s, the maximum paint thickness reduction achieved in a single pass was 93 μm, corresponding to a 22.4% increase in efficiency compared to single-nanosecond laser cleaning. Liu et al. [24] employed finite element simulation to analyze temperature variations and removal depth during the cleaning of rubber deposits from airport runways using nanosecond-continuous lasers. The simulation results indicated that increasing the output energy of the nanosecond-continuous laser led to a progressive increase in removal depth and a corresponding rise in temperature at the laser impact point, thereby enabling the rubber layer to separate from the substrate without causing damage. Dong et al. [25] used a CW laser in combination with nanosecond lasers at varying delay times to clean the oxide film from an aluminum alloy surface, while simultaneously analyzing plasma plumes, surface morphology, and elemental composition. The results indicated that, under a total laser flux of 3630.47 J/cm^2^ (with CW laser flux of 3628.73 J/cm^2^ and nanosecond laser flux of 1.74 J/cm^2^), the removal efficiency increased with longer pulse delays. Existing research on hybrid laser cleaning has predominantly focused on elucidating the underlying mechanisms, investigating the effects of individual parameters on cleaning performance, and comparing the efficacy of hybrid laser modes with that of single-mode lasers. However, systematic studies on the relationships between key parameters of hybrid laser systems (such as CW laser power, pulsed laser power, cleaning speed, and pulse repetition frequency) and surface quality indicators (such as surface roughness and removal rate), as well as their integration into multi-objective optimization frameworks [26,27], remain limited. Therefore, employing a multi-objective optimization approach that integrates laser parameters with cleaning performance to develop an optimized process strategy for hybrid laser cleaning of carbon deposits holds significant practical importance and application potential.

This study investigates the hybrid laser cleaning of carbon deposits on the piston crowns of N52B30 engines, leveraging the combined characteristics of CW and pulsed lasers to perform a multi-objective optimization analysis. This study employs response surface methodology (RSM) to investigate the effects of CW laser power, pulsed laser power, cleaning speed, pulse repetition frequency, and other parameters on surface roughness and carbon residue rate, with the goal of optimizing cleaning performance. The research findings will provide a technical reference for the engineering application of hybrid laser cleaning technology for carbon deposits and promote the broader adoption of laser cleaning in automotive engine maintenance and the remanufacturing of end-of-life components.

## 2. Materials and Methods

### 2.1. Experimental Materials and Equipment

**Figure 1:**
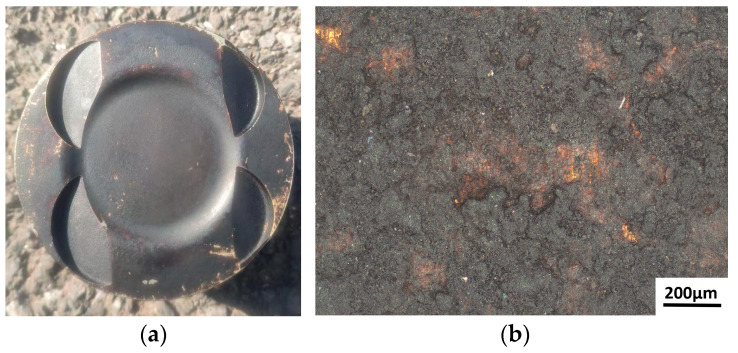
Experimental objects for laser cleaning: (**a**) N52B30 engine piston; (**b**) morphology of carbon deposits on the piston crown.

The experiment utilized a batch of high-mileage BMW N52B30 engine pistons (Bayerische Motoren Werke AG, Munich, Germany) as cleaning specimens. The N52B30 engine features an 85 mm bore, an 88 mm stroke, and a compression ratio of 10.7:1, as depicted in Figure 1. This engine employs an AlSi17 cylinder liner with a high silicon content. To ensure compatibility with the thermal expansion characteristics of the liner, the piston is fabricated from A390 aluminum alloy [28,29], a high-silicon hypereutectic aluminum alloy containing approximately 16–20% silicon. This material selection minimizes variations in piston-to-cylinder wall clearance, thereby enhancing sealing performance and durability. The chemical composition of the A390 alloy is presented in Table 1, while Table 2 illustrates the structure and composition of carbon deposits on the piston crown [30,31].

**Figure 2:**
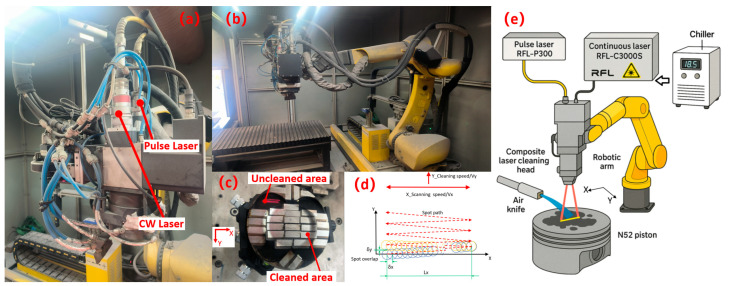
Equipment system and schematic diagram: (**a**) hybrid laser cleaning head; (**b**) robotic arm; (**c**) piston to be cleaned; (**d**) laser spot path; (**e**) schematic diagram of equipment system operation.

As illustrated in Figure 2, the system comprises a CW laser, a pulsed laser, a hybrid laser head, a robotic arm, an air knife, and a water-cooled chiller. The lasers serve as the light sources, while the hybrid laser head directs and adjusts the beam path. The laser head incorporates two collimators, field lenses, and two independent galvanometer sets, which scan the two laser beams reciprocally along the X-direction. The robotic arm governs the Y-direction movement of the laser head, ensuring precise cleaning. The air knife assembly delivers compressed air to shield the treatment area, preventing the ingress of oxides and contaminants, thereby preserving surface cleanliness. Heat dissipation for both lasers and the cleaning head is managed by the water-cooled chiller. Detailed specifications of each component are provided in Table 3.

Position the piston to be cleaned on the workbench (Figure 2c), and enter the process parameters into the computer system and equipment interface (Table 4). The lasers emit beams that are transmitted through the beam delivery system to the scanning mirrors within the laser head. The beams are then focused onto the workpiece surface by focusing lenses to form laser spots. The scanning mirrors control the scanning speed and line width of the laser beams in the X direction, while the robotic arm controls the cleaning speed and direction of the laser beams in the Y direction. The actual trajectory of the laser spots moves along a sawtooth-shaped path (as shown in Figure 2d). Due to the limited number of available pistons, multiple rectangular areas measuring 16 mm × 5 mm were cleaned on each piston under different process parameters.

### 2.2. Experimental Methods

#### 2.2.1. Characterization Method for Surface Roughness and Carbon Residue Rate

To comprehensively evaluate the effectiveness of hybrid laser cleaning, this study employed a confocal laser scanning microscope (CLSM) to acquire high-resolution images of the sample surfaces and to observe changes in surface morphology before and after cleaning. The confocal images enabled quantitative analysis of surface roughness and localized defects, providing a basis for assessing the impact of the cleaning process on the substrate. The microstructure after laser cleaning was observed at high magnification using a scanning electron microscope (SEM, ZEISS EVO18, Carl Zeiss AG, Oberkochen, Germany). An energy-dispersive spectrometer (EDS, Nano X Flash Detector 5010, Bruker Corporation, Billerica, MA, USA) was employed to analyze the elemental distribution and regional composition, allowing for evaluation of the cleaning effectiveness.

**Figure 3:**
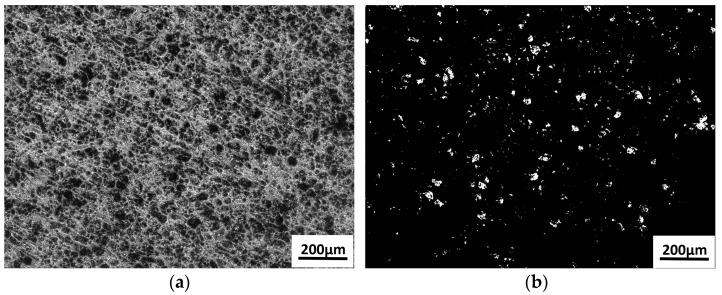
Binary evaluation of carbon residue rate: (**a**) grayscale image; (**b**) inverse binary processing.

To quantitatively assess the effectiveness of carbon deposit removal from the piston crown, this study employed a digital image processing-based analysis method [32]. The digital image of the cleaned area for each experimental group was acquired using the analysis software of CLSM (VK-Analyzer, Keyence Corporation, Osaka, Japan). For example, the following process parameters were applied: CW laser power at 20% of its maximum output, pulsed laser power at 20% of its maximum output, Y-direction cleaning speed of 20 mm/s, pulse repetition frequency of 35 kHz, and X-direction scanning speed of 5000 mm/s. Unless otherwise specified, all laser power percentages mentioned hereafter refer to a proportion of their respective maximum output power. Raw images of the cleaning results were acquired using analysis software, and image processing was performed using Python (3.11.13) in combination with the OpenCV computer vision library (4.12.0) [33,34,35,36]. First, the original image was converted to a grayscale image (Figure 3a), and a 5 × 5 Gaussian kernel was applied for noise reduction to minimize interference from surface microstructures. Next, an inverse binarization process was applied with a threshold of 50 pixels (Figure 3b), wherein carbon deposit regions appeared as white pixels (pixel value 255) and clean regions as black pixels (pixel value 0). The proportion of white pixels in the binary image was then calculated (Formula (1)). This method effectively mitigates the subjective bias inherent in traditional visual inspection by providing digital quantitative analysis, offering high repeatability along with intuitive results.

Carbon residue rate (RC) calculation equation:

(1) $$\mathrm{RC}=\frac{\mathrm{Number}\;\mathrm{of}\;\mathrm{white}\;\mathrm{pixels}}{\mathrm{Total}\;\mathrm{number}\;\mathrm{of}\;\mathrm{pixels}}\times 100\%$$

In Equation (1), RC represents the proportion of the carbon residue area (i.e., the carbon residue rate). White pixels correspond to carbon residue regions in the binarized image (defined in the code as pixels with a value of 255 through threshold segmentation). The total number of pixels refers to the image resolution (e.g., the product of image width and height).

#### 2.2.2. Single-Factor Experiment

1. Effect of X-Direction Scanning Speed on Surface Roughness (Sa)

Under the conditions of pulse repetition frequency 30 kHz, Y-direction cleaning speed 25 mm/s, pulsed laser power 75%, and CW laser power 25%, a single-factor experiment was conducted on the X-direction scanning speed [37], with values of 1000 mm/s, 3000 mm/s, 5000 mm/s, 7000 mm/s, and 9000 mm/s, and the surface roughness (Sa) was measured.

2. Effect of Pulse Repetition Frequency on Surface Roughness (Sa)

Under conditions of Y-direction cleaning speed of 25 mm/s, pulsed laser power of 75%, and CW laser power of 25%, the optimal X-direction scanning speed was selected, and a single-factor experiment was conducted on the pulse repetition frequency, with values of 10 kHz, 20 kHz, 30 kHz, 40 kHz, and 50 kHz, and the surface roughness (Sa) was measured.

3. Effect of Y-Direction Cleaning Speed on Surface Roughness (Sa)

Under conditions of pulsed laser power 75% and CW laser power 25%, with the X-direction scanning speed and pulse repetition frequency optimized, a single-factor experiment was conducted on the Y-direction cleaning speed, selecting values of 5 mm/s, 15 mm/s, 25 mm/s, 35 mm/s, and 45 mm/s, and the surface roughness (Sa) was measured.

4. Effect of Pulsed Laser Power on Surface Roughness (Sa)

Under conditions of CW laser power 25%, the optimal values for X-direction scanning speed, pulse repetition frequency, and Y-direction cleaning speed were selected, and a single-factor experiment was conducted on the pulsed laser power, with values of 55%, 65%, 75%, 85%, and 95% selected, and the surface roughness (Sa) was measured.

5. Effect of CW Laser Power on Surface Roughness (Sa)

Under optimal conditions for all other parameters, a single-factor experiment was conducted on the CW laser power, selecting values of 5%, 15%, 25%, 35%, and 45%, and the surface roughness (Sa) was measured.

#### 2.2.3. Plackett–Burman Design

The Plackett–Burman design was implemented using Design-Expert software (version 12) [38,39], with 12 experimental runs, to evaluate the influence of five factors: CW laser power (A, %), pulsed laser power (B, %), Y-direction cleaning speed (C, mm/s), pulse repetition frequency (D, kHz), and X-direction scanning speed (E, mm/s). Surface roughness (Sa) was selected as the response variable to assess the significance of each factor (Table 5).

#### 2.2.4. The Steepest Ascent Test

The steepest ascent test [40] utilizes the gradient direction of the response surface to guide the experimental path, with the step size determined by the relative magnitude of the effects of each significant factor. This approach enables rapid convergence toward the region of maximum response. Based on the results of the Plackett–Burman design, the four most significant factors—CW laser power, pulsed laser power, Y-direction cleaning speed, and pulse repetition frequency—were selected for the steepest ascent test.

#### 2.2.5. The Box–Behnken Design

The Box–Behnken design (BBD) [41,42,43] is a widely used response surface methodology (RSM) for process optimization. In this study, Design-Expert software was employed to perform a four-factor, three-level BBD experiment (Table 6) based on the results of the steepest ascent test. Surface roughness (Sa) and carbon residue rate (RC) were selected as the response variables to identify the optimal process parameters for multi-objective optimization.

It is particularly important to note that the surface roughness of the piston crown has a significant influence on the performance of internal combustion engines, directly affecting combustion efficiency, power output, fuel economy, and emissions. A moderate surface roughness (Sa: 0.4–1 μm) enhances in-cylinder turbulence [28,31], promotes fuel–air mixing, and improves combustion stability, resulting in a 1–3% increase in power output and a 1–2% reduction in fuel consumption. Moreover, surface roughness influences thermal stress distribution and the propensity for carbon buildup, both of which impact the durability of the piston. Therefore, this study prioritizes optimizing surface roughness while also considering carbon residue rates to achieve the best possible overall engine performance.

## 3. Results and Analysis

### 3.1. Results and Analysis of Single-Factor Experiment

**Figure 4:**
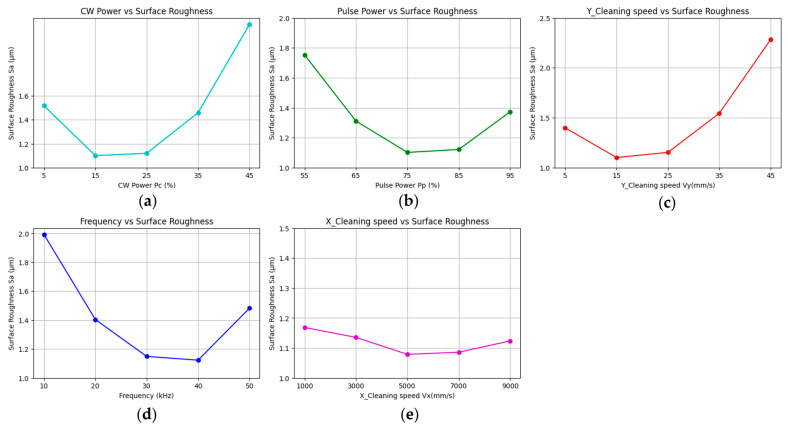
Selection of factors for single-factor experiment: (**a**) CW laser power; (**b**) pulsed laser power; (**c**) Y_Scanning speed; (**d**) repetition frequency; (**e**) X_Scanning speed.

Single-factor experiments were conducted to evaluate the effects of various process parameters on surface roughness (Sa) within a defined range. As shown in the factor-response plots (Figure 4), the optimal X-direction scanning speed corresponded to a minimum Sa value at 5000 mm/s; pulse repetition frequency yielded its lowest Sa at 40 kHz; Y-direction cleaning speed achieved optimal results at 15 mm/s; pulsed laser power was optimal at 75%; and CW laser power produced the best outcome at 15%.

### 3.2. Results of the Plackett–Burman Design

Building upon the results of the single-factor experiments, a Plackett–Burman design comprising 12 trials was employed, with surface roughness (Sa) selected as the response variable. The experimental design and corresponding results are presented in Table 7.

**Figure 5:**
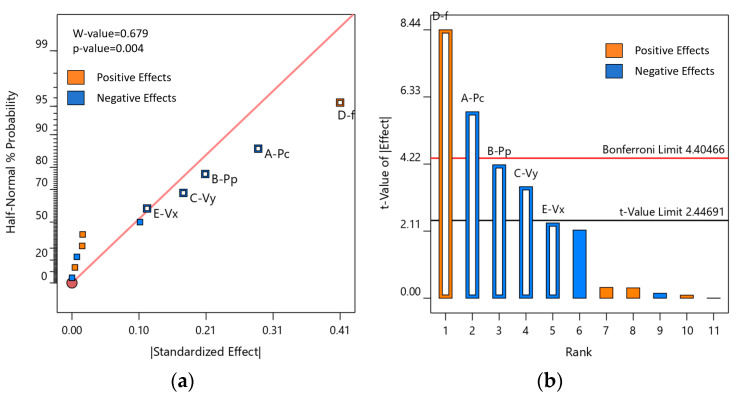
Model effects diagram: (**a**) half-normal plot; (**b**) Pareto chart.

An analysis of variance (ANOVA) was performed on the experimental data. The results of the Plackett–Burman design were further analyzed using the Lenth method to identify statistically significant effects, resulting in a semi-normal probability plot (Figure 5a) and a Pareto chart of standardized effects (Figure 5b). As shown in Figure 5a, the standardized effect points of factors D, A, B, and C deviate substantially from the fitted line, indicating that these are statistically significant factors influencing surface roughness (*p* < 0.05). Therefore, the primary factors significantly affecting surface roughness (Sa) are the CW laser power (Pc), pulsed laser power (Pp), Y-direction cleaning speed (Vy), and pulse repetition frequency (f). The standardized effects of the remaining factors are comparatively minor. The Pareto chart of standardized effects (Figure 5b) further confirms both the magnitude and statistical significance of these effects, with factors A, B, C, and D all exceeding the t-value threshold for significance.

As shown in Table 8, the model *p*-value is 0.0004 (<0.05), indicating that the model is statistically significant and therefore reliable. This implies that the model provides a good fit across the entire regression region. The coefficient of determination (R^2^) of 0.9592 indicates a strong correlation between the predicted and experimental values. The adjusted coefficient of determination (R^2^adj) of 0.9252 indicates that 92.52% of the variability in the experimental data is explained by the regression model. The predicted R^2^ value is 0.8367, demonstrating reasonable agreement with the adjusted R^2^ value of 0.9252 (with a difference of less than 0.2). In general, a lower coefficient of variation (C.V.) indicates higher experimental reliability and precision. A C.V. value of 5.2% suggests that the Plackett–Burman design exhibits good reliability and precision. Precision, defined as the ratio of effective signal to noise (Adeq Precision), is considered acceptable when greater than 4.0. In this experiment, the Adeq Precision reached 17.9610, indicating a highly adequate signal-to-noise ratio. Multiple regression analysis of the data yielded the following regression equation:

Sa = 2.06625 − 0.014358A − 0.010275B − 0.008592C + 0.020675D − 0.000029E(2)

The regression Equation (2) shows that the partial regression coefficient of factor D is 0.020675, indicating that factor D exerts a positive influence on surface roughness (Sa). Specifically, decreasing factor D leads to a reduction in surface roughness (Sa). Factors A, B, and C exhibit negative effects, meaning that increasing these factors results in lower surface roughness (Sa). Subsequent optimization focuses on CW laser power, pulsed laser power, Y-direction cleaning speed, and pulse repetition frequency.

### 3.3. Analysis of the Results of the Steepest Ascent Test

The steepest ascent test necessitates a comprehensive evaluation of factors such as cost and operational workload. The X-direction scanning speed was fixed at the optimal value of 5000 mm/s, as determined from the single-factor experiments, and the ascent was performed using a step size of 0.5 times the original increment (corresponding to a tolerance of 5). The experimental design and corresponding results are presented in Table 9.

As shown in Table 9, sample 2 exhibited the lowest surface roughness (Sa), and therefore, its parameter values were selected as the center point for the subsequent response surface experiment.

### 3.4. Analysis of the Results of the Box–Behnken Design

Based on the results of the steepest ascent test, a Box–Behnken experimental design was implemented, using the parameter values of sample 2 as the central point. Four factors—CW laser power, pulsed laser power, Y-direction cleaning speed, and pulse repetition frequency—were chosen as independent variables. A four-factor, three-level experiment was designed according to the Box–Behnken methodology, with surface roughness (Sa) and carbon residue rate (RC) as the response variables. Multi-objective optimization was performed using the response surface methodology (RSM). The experimental design and corresponding results are presented in Table 10.

#### 3.4.1. Analysis of Surface Roughness (Sa) Using the Box–Behnken Design

Through second-order multiple regression analysis of the experimental data, the following quadratic polynomial model was derived:

Sa = 8.75992 − 0.1173A − 0.156717B − 0.042617C − 0.013817D + 0.00065AB − 0.00021AC − 0.00014AD − 0.00006BC − 0.00108BD − 0.00029CD + 0.001882A^2^ + 0.001167B^2^ + 0.001722C^2^ + 0.001547D^2^ (3)

The multiple correlation coefficient of Equation (3) is R^2^ = 0.9635, indicating that the model provides an excellent fit to the experimental data. The experimental results can thus be effectively analyzed using this regression model. The results of the variance analysis (ANOVA) for the response surface experiment are presented in Table 11. As shown in the table, the model is highly significant (*p* < 0.05), whereas the lack-of-fit and other non-significant terms exhibit *p*-values greater than 0.05. The coefficient of determination R^2^ = 0.9635 and the adjusted R^2^ = 0.9208 indicate that the model exhibits strong predictive capability and explains 92.08% of the variability in the response values. This regression model is therefore suitable for analyzing and predicting surface roughness (Sa). As detailed in Table 11, the main effects of factors B and C significantly influence surface roughness (Sa), whereas factors A and D do not demonstrate statistical significance. Among the interaction effects, only AB and BD show significant impacts on Sa, with other interactions being non-significant. Additionally, the quadratic terms A^2^, B^2^, C^2^, and D^2^ significantly affect Sa, indicating that the factor–response relationship is inherently nonlinear. According to the F-values, the factors’ relative influence on surface roughness can be ranked as follows: C > B > A > D, corresponding, respectively, to Y-direction cleaning speed, pulsed laser power, CW laser power, and pulse repetition frequency.

**Figure 6:**
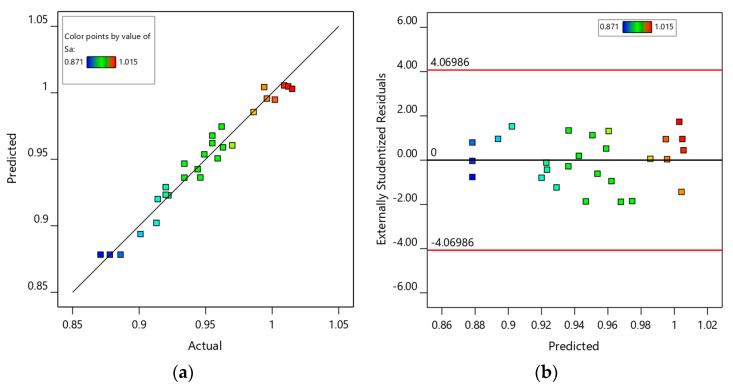
Surface roughness model diagnostic chart: (**a**) chart of SA model predicted values versus experimental values; (**b**) chart of SA model residuals versus predicted values.

A regression model was employed to predict surface roughness (Sa) across various parameter settings. To facilitate observation of the model prediction results, predicted values were plotted against experimental measurements (Figure 6a). The data points cluster closely around the 45° reference line, demonstrating a strong correlation between predicted and actual values, with negligible deviations. Figure 6b illustrates the distribution of residuals from the model predictions. For a robust model, the majority of prediction errors should lie within ±2 standard deviations (SD). As depicted, all residuals fall within this ±2 SD confidence interval, confirming the reliability of the model.

#### 3.4.2. Analysis of Carbon Residue Rate (RC) Using the Box–Behnken Design

Through second-order multiple regression analysis of the experimental data, the following quadratic polynomial model was derived:

RC = 0.588536 + 0.037102A − 0.010968B + 0.02968C − 0.037288D − 0.000409AB − 0.00042AC + 0.000043AD − 0.000096BC + 0.000535BD − 0.000323CD(4)

The multiple correlation coefficient for Equation (4) is R^2^ = 0.9361, indicating a strong model fit to the experimental data. This equation can thus be reliably used to analyze the results. The response surface analysis outcomes are summarized in Table 12.

As shown in Table 12, the model is highly significant (*p* < 0.05) and the lack of fit is not significant (*p* > 0.05). The coefficient of determination R^2^ = 0.9361 and the adjusted coefficient of determination R^2^ adj = 0.8962 indicate that the model fits well and can explain 89.62% of the variation in the response values. This model can be used to analyze and predict the residue rate of carbon deposits (RC). As shown in Table 12, the main factors A, B, and C have a significant effect on the carbon residue rate (RC), while D has no significant effect. The interaction terms AB, AC, BD, and CD have a significant effect on the carbon residue rate (RC), while AD and BC have no significant effect. The quadratic terms A^2^, B^2^, C^2^, and D^2^ have no significant effect on the carbon residue rate (RC). This indicates that the relationship between the factors and the response value is not a simple linear relationship. Based on the F-value, the influence of the factors is ranked as follows: A > B > C > D, corresponding, respectively, to CW laser power, pulsed laser power, Y-direction cleaning speed, and pulse repetition frequency.

**Figure 7:**
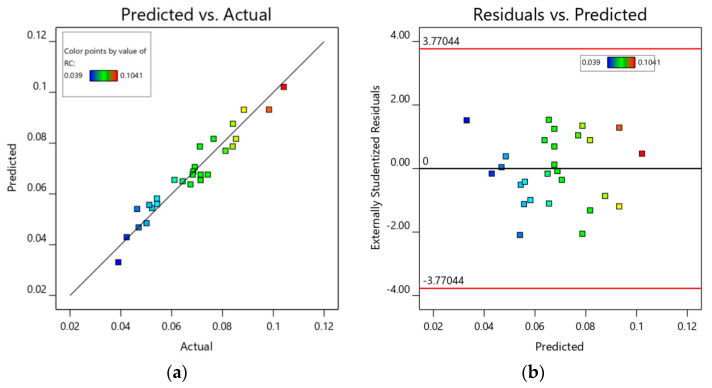
Carbon residue rate model diagnostic chart: (**a**) chart of RC model predicted values versus experimental values; (**b**) chart of RC model residuals versus predicted values.

A regression model was employed to predict carbon residue rate (RC) across various parameter settings. To facilitate observation of the model prediction results, predicted values were plotted against experimental measurements (Figure 7a). The data points cluster closely around the 45° reference line, demonstrating a strong correlation between predicted and actual values, with negligible deviations. Figure 7b illustrates the distribution of residuals from the model predictions. For a robust model, the majority of prediction errors should lie within ±2 standard deviations (SD). As depicted, all residuals fall within this ±2 SD confidence interval, confirming the reliability of the model.

**Figure 8:**
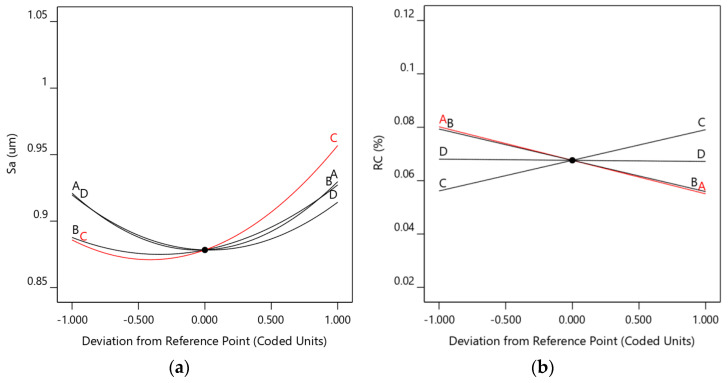
Diagram of perturbation (**a**) in surface roughness; (**b**) in carbon residue rate.

Figure 8a shows the surface roughness perturbation diagram, indicating that the Y-direction cleaning speed has the most significant effect on surface roughness, followed by the pulsed laser power. As the cleaning speed increases, the surface roughness first decreases and then increases. This is primarily due to the fact that an excessively low cleaning speed causes laser energy to over-accumulate, forming noticeable ablation marks on the substrate surface, thereby increasing surface roughness. Conversely, an excessively high cleaning speed reduces the spot overlap rate, weakening the thermal accumulation effect of the laser, which fails to effectively remove carbon deposits, resulting in an uneven carbon deposit layer and similarly increasing surface roughness. Similarly, while keeping other parameters constant, as the pulsed laser power increases, the surface roughness first decreases slowly and then rises rapidly. The primary reason is that as the laser energy density increases, the carbonized layer on the surface is rapidly sublimated and peeled off. As the inner oxide layer reaches its boiling point and vaporizes, the vapor above the substrate absorbs laser energy, generating a plasma effect. The molten substrate is impacted, forming pits that increase surface roughness.

**Figure 9:**
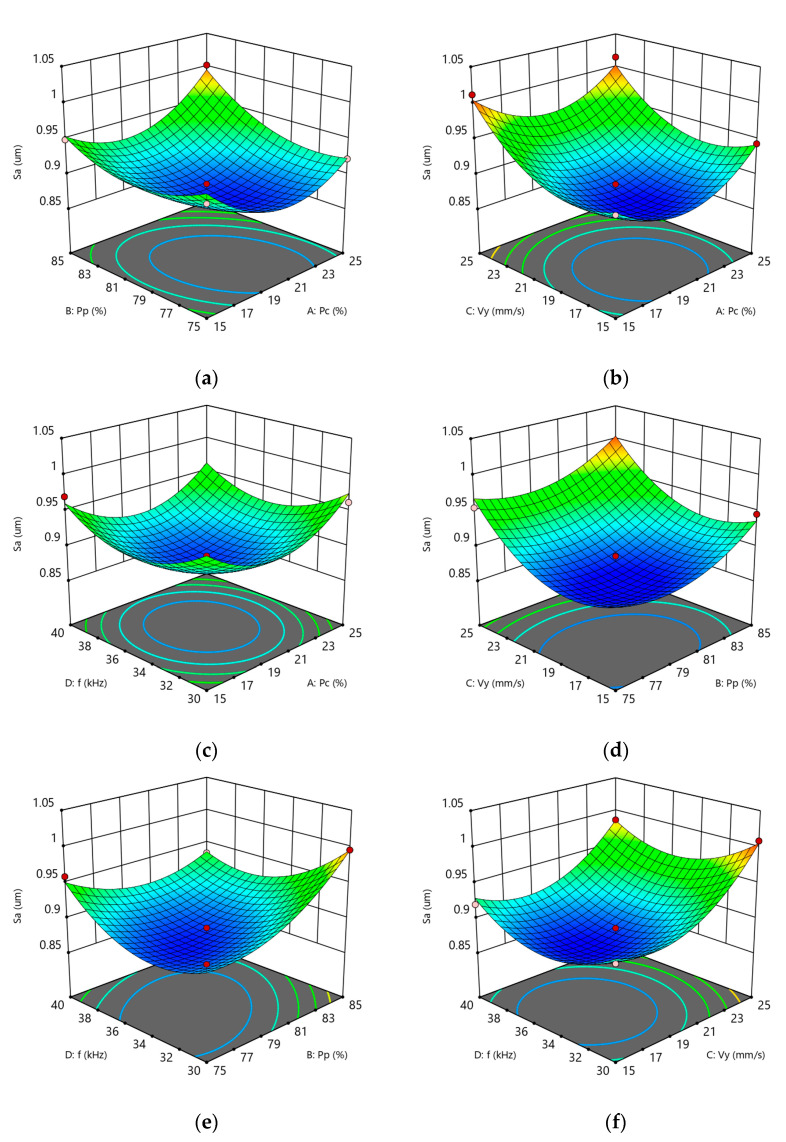
Response surface plots for each interaction term in Sa: (**a**) AB response surface plot roughness; (**b**) AC response surface plot; (**c**) AD response surface plot; (**d**) BC response surface plot; (**e**) BD response surface plot; (**f**) CD response surface plot.

**Figure 10:**
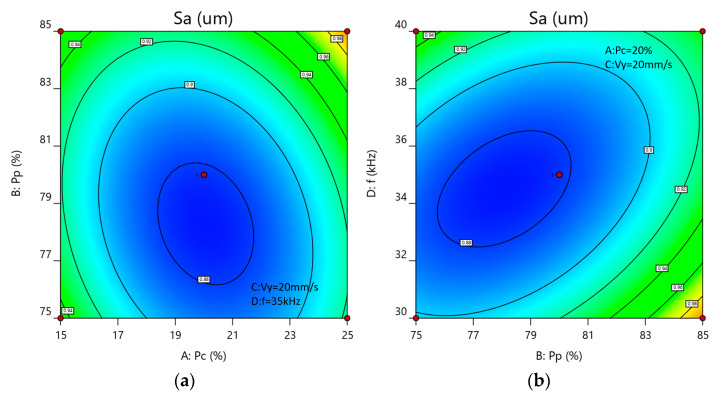
The impact of significant interaction terms on surface roughness: (**a**) AB contour plot; (**b**) BD contour plot.

Figure 9 shows the influence of the interaction between process parameters on surface roughness. According to the response surface diagram of the interaction between process parameters, the surface roughness after laser cleaning is significantly affected by the interaction between the CW laser power (factor A) and the pulsed laser power (factor B), as well as between the pulsed laser power (factor B) and the pulse repetition frequency (factor D), consistent with the results of the ANOVA. Analysis of the contour plots for AB and BD indicates that the synergistic interaction between pulsed laser and CW laser parameters significantly influences surface roughness (Sa) during hybrid laser cleaning of carbon deposits. Figure 10a shows that when Pc is controlled within the range of 19–21% and Pp is controlled within the range of 76–80% (with Vy fixed at 20 mm/s and f at 35 kHz), an elliptical low-roughness core zone is formed. This confirms that the thermal softening effect of CW laser (at approximately 20%) significantly reduces the bond strength between carbon deposits and the substrate, providing a uniform thermal field for the pulsed laser (at approximately 78%) to peel off carbon deposits, thereby effectively protecting the substrate surface during carbon removal. Figure 10b further reveals the regulatory mechanism of the pulse repetition frequency (f): when Pp is stable at 76–80% (fixed Vy = 20 mm/s; Pc = 20%), f shows a significant optimization effect in the range of 33–36 kHz, indicating that this frequency band can ensure sufficient pulse energy density while avoiding energy accumulation caused by high frequencies.

Figure 8b shows the perturbation diagram of the carbon residue rate. It can be seen that the CW laser power has the most significant effect on surface roughness, followed by the pulsed laser power. As the continuous and pulsed laser power increases, the carbon residue rate (RC) on the piston top decreases. The primary reason is that, under conditions of continuously increasing laser power, both the laser energy density and the laser power density increase, leading to a greater amount of laser energy absorbed per unit area of the base material. This results in stronger ablation and impact effects on the carbon deposit layer, thereby increasing the carbon removal thickness. The carbonized layer and oxide layer rapidly sublimate and vaporize, causing the carbon residue rate (RC) to decrease. As the scanning speed increases, the spot overlap rate decreases, the gap rate increases, and the thermal accumulation effect of the laser weakens, making it ineffective in removing carbon deposits. As a result, the carbon residue rate (RC) increases. Increasing the pulse repetition frequency reduces the energy density of a single pulse but simultaneously increases the cumulative laser energy per unit area. Therefore, changes in pulse repetition frequency have a limited impact on carbon removal.

**Figure 11:**
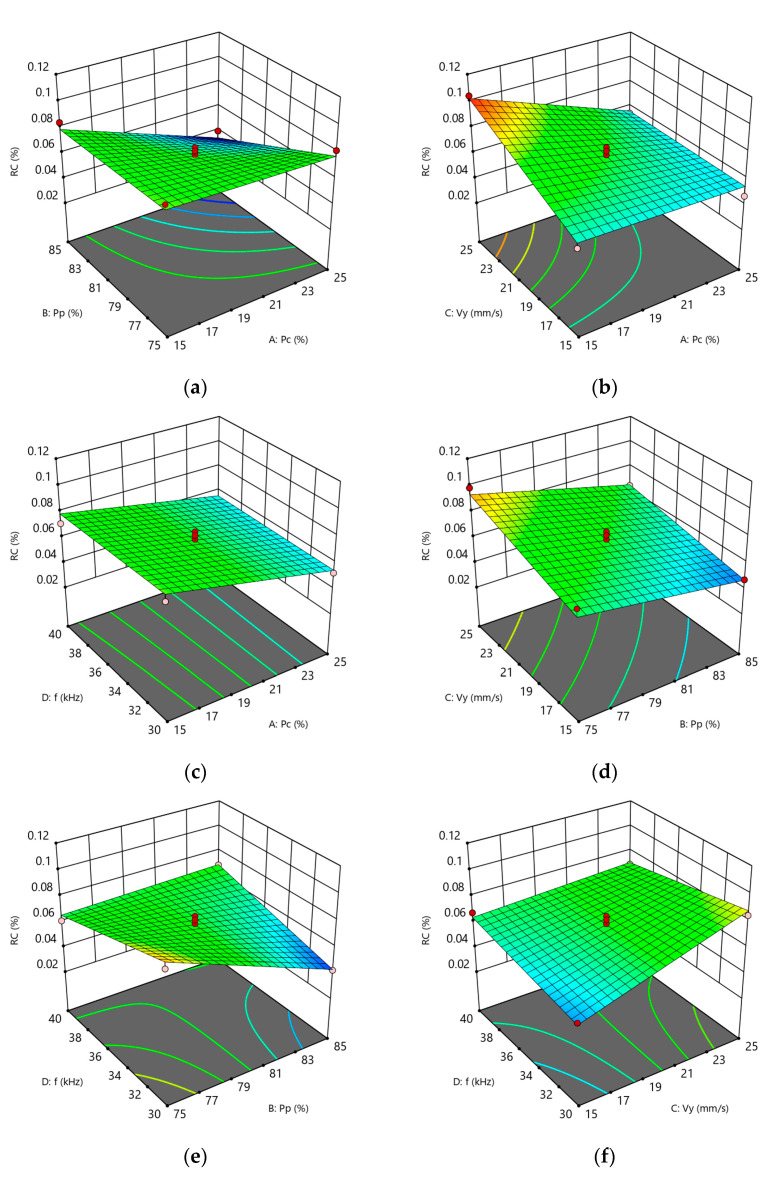
Response surface plots for each interaction term in RC: (**a**) AB response surface plot roughness; (**b**) AC response surface plot; (**c**) AD response surface plot; (**d**) BC response surface plot; (**e**) BD response surface plot; (**f**) CD response surface plot.

Figure 11 illustrates the effects of interactions among various process parameters on the carbon residue rate (RC), highlighting that interactions between AB, AC, BD, and CD exhibit a more pronounced influence. Response surface analysis suggests that achieving a lower carbon residue rate can be facilitated by moderately reducing the Y-direction cleaning speed and pulse repetition frequency, while concurrently increasing both CW and pulsed laser power. However, these adjustments must be balanced with surface quality constraints to enable effective multi-objective optimization.

### 3.5. Multi-Objective Optimization of Process Parameters

Based on the above analysis, the predictive performance of both models for surface roughness (Sa) and carbon residue rate (RC) satisfies the established requirements. Therefore, the two regression models can be employed to optimize the quality of the hybrid laser cleaning process. The optimization objectives for hybrid laser cleaning of carbon deposits are summarized in Table 13. Surface roughness (Sa) is optimized toward the lower bound of the target range (0.4–1.0 μm) [28,31], while carbon residue rate (RC) is optimized toward the lower bound of the target range (0–0.05) [6]. This approach aims to achieve the surface roughness target required for the optimal piston performance while maximizing the carbon removal efficiency.

The Numerical Optimization module of Design-Expert software was employed to solve the optimization objectives defined in Table 13. The optimal process parameters for hybrid laser cleaning of carbon deposits were determined as follows: CW laser power at 20.86%; pulsed laser power at 83.36%; Y-direction cleaning speed: 15.00 mm/s; pulse repetition frequency: 31.54 kHz; resulting surface roughness (Sa): 0.947 μm; carbon residue rate (RC): 3.67%. To verify the reliability of the predictive model, cleaning experiments were conducted on three distinct regions of the piston top under the optimized process parameters. The corresponding surface roughness and carbon residue rates were measured, and the results are presented in Table 14. All measured values fell within the predicted confidence intervals of the model, with relative errors below 10%, thereby satisfying typical industrial accuracy requirements. These findings further confirm the effectiveness and robustness of the model in guiding multi-objective parameter optimization for hybrid laser cleaning.

It should be noted that the pistons used in this study were sourced from multiple engines with varying operating conditions, leading to differences in the morphology, thickness, and adhesion state of carbon deposits across different regions of different pistons. Additionally, variations in energy coupling during laser cleaning and potential measurement errors may contribute to slight discrepancies between the predicted and measured values. As such, minor deviations are considered reasonable. Moreover, the prior Box–Behnken experimental design already encompassed carbon deposits from multiple regions across different pistons, and the validation experiments were likewise performed on varied areas. Therefore, the final optimal process parameters obtained have a certain degree of adaptability to the cleaning objects, providing a reliable basis for practical engineering applications.

**Figure 12:**
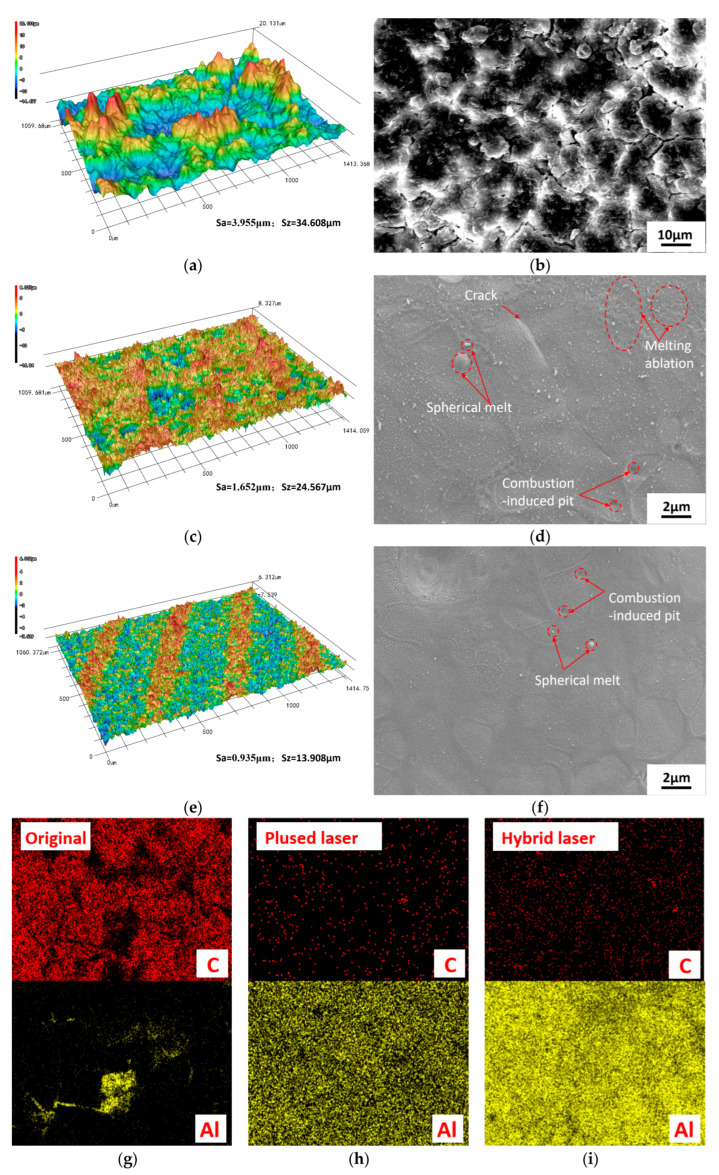
Three-dimensional surface morphology with roughness values, SEM surface topography, and elemental distribution: (**a**) uncleaned area—3D morphology and surface roughness; (**b**) uncleaned area—SEM surface topography; (**c**) single-mode pulsed laser cleaned area—3D morphology and surface roughness; (**d**) single-mode pulsed laser cleaned area—SEM surface topography; (**e**) sample 2—3D morphology and surface roughness; (**f**) sample 2—SEM surface topography; (**g**) uncleaned area—EDS elemental distribution of major elements (carbon and aluminum); (**h**) single-mode pulsed laser cleaned area—EDS elemental distribution of major elements (carbon and aluminum); (**i**) sample 2—EDS elemental distribution of major elements (carbon and aluminum).

As shown in Figure 12, confocal microscopy three-dimensional morphology and roughness analysis, SEM microstructure analysis, and EDS element distribution analysis were performed on the original carbon deposit surface, the single-mode pulsed laser cleaning area (pulsed laser power at 100%), and the hybrid laser cleaning area with optimal process parameters (Sample 2). The results show that the surface of the hybrid laser cleaning area appears smoother and more uniform, with no signs of ablation or cracks. Only some residual spherical carbon particles can be observed, and the overall base metal is clearly exposed. The single-mode pulsed laser cleaning area not only shows more residual carbon deposits compared to the area cleaned under the optimal hybrid laser parameters but also exhibits cracks and ablation zones, resulting in a higher surface roughness. These differences are further supported by the EDS analysis, particularly in the distributions of carbon (C) and aluminum (Al) elements.

From the perspective of the hybrid laser cleaning mechanism [23,24,25], the optimal parameter combination achieves a superior cleaning performance due to the synergistic effects of the two laser sources. The CW laser, operating at approximately 20%, provides uniform and moderate thermal softening of the carbon deposit layer, thereby reducing the interfacial bond strength between the deposits and the substrate and offering thermal assistance for subsequent removal. The pulsed laser, on the other hand, delivers high peak energy that rapidly vaporizes and ejects the softened carbon deposits, significantly improving cleaning efficiency while minimizing damage to the substrate. When applied at appropriate repetition frequencies and scanning speeds, the two lasers maintain a dynamic balance in energy delivery and thermal accumulation, effectively achieving low surface roughness and minimal carbon residue. This demonstrates the advantage of hybrid laser systems in enabling multi-mechanism synergistic cleaning processes.

## 4. Conclusions

This study developed an efficient laser cleaning process based on the synergistic interaction between CW and pulsed lasers to meet the carbon deposit removal requirements on the piston crowns of the N52B30 engine. The process was optimized using the response surface methodology (RSM) for multi-objective optimization. The experimental results revealed that the Y-direction cleaning speed and pulsed laser power had the most significant effects on surface roughness (with F-values of 112.58 and 34.85, respectively), while CW laser power was the primary factor influencing the carbon residue rate (F-value of 57.74). Notably, a significant interactive effect was observed among these three parameters, indicating a synergistic regulation of cleaning quality. By employing a Plackett–Burman design to identify significant factors, followed by a steepest ascent method to determine the optimization path, and finally, applying a Box–Behnken response surface design, the optimal parameter set was obtained: CW laser power of 20.86% (625.8W), pulsed laser power of 83.36% (250.08W), Y-direction cleaning speed of 15.00 mm/s, and pulse repetition frequency of 31.54 kHz. Under these optimized conditions, the surface roughness (Sa) was reduced to 0.947 μm, and the carbon residue rate (RC) decreased to 3.67%, fulfilling the surface quality requirements for in-service internal combustion engine components.

It is worth emphasizing that the optimal parameter combination is not merely the mathematical result of response surface optimization, but also reflects the optimization of physical mechanisms and energy matching principles in the hybrid laser cleaning process: CW lasers provide a stable macro thermal field to soften the carbon deposit layer, while pulsed lasers achieve rapid removal of carbon deposits with high peak energy. Under appropriate hybrid laser power ratios, repetition frequencies, and cleaning speeds, a synergistic mechanism is formed that enables efficient removal of carbon deposits while preserving the integrity of the substrate surface. The methodological innovation of this study is not only reflected in the introduction of hybrid laser cleaning technology in the field of carbon deposit cleaning, but also in the optimization of process parameters for hybrid laser cleaning of carbon deposits through a systematic experimental design. The established optimization process can be extended to the development of cleaning processes for other carbon-deposited components in engines, particularly for large-scale, high-precision carbon removal tasks in remanufacturing scenarios. This provides practical pathways and theoretical support for the expansion of laser cleaning in high-end maintenance and green manufacturing.

## Figures and Tables

**Table 1:** The chemical composition of the A390 alloy.

Major Alloying Elements (wt%)				Minor Alloying Elements (wt%)					Al Content (wt%)
Si	Cu	Mg	Fe	Mn	Zn	Cr	Ni	Ti	
19.14	3.28	0.62	0.42	0.05	0.04	0.01	0.01	0.014	76.443

**Table 2:** The structure and composition of carbon deposits on the piston crown.

Carbon Deposit Layer	Thickness Range (μm)	Main Components
Surface layer (Loose layer)	10–50	Hydrocarbons (HC); Resin and asphalt
Intermediate layer (Dense layer)	50–150	Graphitic carbon (C); Metal oxides (Fe_2_O_3_, Al_2_O_3_); Sulfides (SOx)
Base layer (Oxidized/sulfurized layer)	5–20	Metal particles (Fe, Ni, Cr); Nitrogen oxides (NOx); Sulfides (FeS, CuS)

**Table 3:** Device model.

Equipment	Model
Pulsed lasers	RFL-P300 (Raycus Fiber Laser Technologies Co., Ltd., Wuhan, China)
CW fiber lasers	RFL-C3000 (Raycus Fiber Laser Technologies Co., Ltd., Wuhan, China)
Smoke purifiers	DB2400 (S&A Industrial Equipment Co., Ltd., Guangzhou, China)
Laser cleaning head	FH1-ACiF1-2K0300DZ2-23M01 (Han’s Laser Technology Industry Group Co., Ltd., Shenzhen, China)
Laser water cooler	TFLW-3000WDR (Tongfei Technology Co., Ltd., Suzhou, China)
Robotic arm	FANUC M-20ID (FANUC Corporation, Yamanashi, Japan)

**Table 4:** Ranges of the main parameters of the laser cleaning platform.

Pulsed Laser		Semiconductor Laser	
Power/W	0–300	Power/W	0–3000
X-Scanning speed/mm·s^−1^	5000	X_Scanning speed/mm·s^−1^	5000
Spot diameter/μm	350	Spot diameter/μm	2100
Line width/mm	16	Line width/mm	16
Frequency/kHz	10–50	Frequency/kHz	2
Pulse width/ns	150	Duty cycle/%	100
Center wavelength/nm	1064 ± 5	Center wavelength/nm	1080 ± 5
Core diameter/μm	100	Core diameter/μm	600
Y_Cleaning speed/mm·s^−1^		0–2400	
Defocus amount/mm		0	
Axial moving speed of laser head/mm·s^−1^		19.5	
Spot spacing/mm		1	

**Table 5:** Factor levels in Plackett–Burman design.

Name	Units	Low	High
A:Pc	%	5	25
B:Pp	%	65	85
C:Vy	mm/s	5	25
D:f	kHz	30	50
E:Vx	mm/s	3000	7000

**Table 6:** Factor levels in Box–Behnken.

Name	Units	Level		
		−1	0	+1
A:Pc	%	15	20	25
B:Pp	%	75	80	85
C:Vy	mm/s	15	20	25
D:f	kHz	30	35	40

**Table 7:** Plackett–Burman experimental designs and results.

Std	Run	A:Pc	B:Pp	C:Vy	D:f	E:Vx	Sa
		%	%	mm/s	kHz	mm/s	μm
10	1	5	85	25	50	3000	1.753
4	2	5	85	5	50	7000	1.88
11	3	25	65	25	50	7000	1.705
5	4	5	65	25	30	7000	1.495
6	5	5	65	5	50	3000	2.329
1	6	25	85	5	50	7000	1.61
3	7	25	65	25	50	3000	1.765
9	8	25	85	25	30	3000	1.145
2	9	5	85	25	30	7000	1.423
8	10	25	85	5	30	3000	1.374
7	11	25	65	5	30	7000	1.341
12	12	5	65	5	30	3000	1.783

**Table 8:** Plackett–Burman experimental factors, their levels, and significance evaluation.

Source	Sum of Squares	df	Mean Square	F-Value	*p*-Value	
Model	1.02	5	0.2032	28.20	0.0004	significant
A-Pc	0.2474	1	0.2474	34.34	0.0011	
B-Pp	0.1267	1	0.1267	17.58	0.0057	
C-Vy	0.0886	1	0.0886	12.30	0.0127	
D-f	0.5129	1	0.5129	71.20	0.0002	
E-Vx	0.0403	1	0.0403	5.59	0.0560	
Residual	0.0432	6	0.0072			
Cor Total	1.06	11				

R^2^ = 0.9592; Predicted R^2^ = 0.8367; C.V.% = 5.20; Adjusted R^2^ = 0.9252; Adeq Precision = 17.9610.

**Table 9:** Experimental design and results of the steepest ascent method.

Sample	A	B	C	D	Sa
	%	%	mm/s	kHz	μm
1	15	75	15	40	1.020
2	20	80	20	35	0.878
3	25	85	25	30	1.145
4	30	90	30	25	1.821
5	35	95	35	20	2.906

**Table 10:** Box–Behnken experimental designs and results.

Std	Run	A:Pc	B:Pp	C:Vy	D:f	Sa	RC
		%	%	mm/s	kHz	μm	
12	1	25	80	20	40	0.955	0.0512
22	2	20	85	20	30	0.996	0.0423
15	3	20	75	25	35	0.955	0.0983
14	4	20	85	15	35	0.946	0.047
17	5	15	80	15	35	0.92	0.0542
21	6	20	75	20	30	0.913	0.0884
25	7	20	80	20	35	0.886	0.0742
13	8	20	75	15	35	0.901	0.0714
11	9	15	80	20	40	0.97	0.0711
5	10	20	80	15	30	0.914	0.0501
26	11	20	80	20	35	0.878	0.0683
3	12	15	85	20	35	0.949	0.084
20	13	25	80	25	35	1.015	0.0543
9	14	15	80	20	30	0.963	0.0765
23	15	20	75	20	40	0.959	0.0611
19	16	15	80	25	35	1.012	0.1041
4	17	25	85	20	35	1.002	0.039
8	18	20	80	25	40	0.986	0.0691
7	19	20	80	15	40	0.92	0.0674
6	20	20	80	25	30	1.009	0.0841
18	21	25	80	15	35	0.944	0.0464
1	22	15	75	20	35	0.934	0.0853
10	23	25	80	20	30	0.962	0.0523
16	24	20	85	25	35	0.994	0.0643
27	25	20	80	20	35	0.871	0.0714
2	26	25	75	20	35	0.922	0.0812
24	27	20	85	20	40	0.934	0.0685

**Table 11:** Analysis of variance (ANOVA) for the quadratic model in the Box–Behnken design (Sa).

Source	Sum of Squares	df	Mean Square	F-Value	*p*-Value	
Model	0.0425	14	0.0030	22.61	<0.0001	significant
A-Pc	0.0002	1	0.0002	1.68	0.2196	
B-Pp	0.0047	1	0.0047	34.85	<0.0001	
C-Vy	0.0151	1	0.0151	112.58	<0.0001	
D-f	0.0001	1	0.0001	0.6756	0.4271	
AB	0.0011	1	0.0011	7.86	0.0159	
AC	0.0001	1	0.0001	0.8208	0.3828	
AD	0.0000	1	0.0000	0.3648	0.5571	
BC	9.0 × 10^−6^	1	9.0 × 10^−6^	0.0670	0.8001	
BD	0.0029	1	0.0029	21.71	0.0006	
CD	0.0002	1	0.0002	1.57	0.2347	
A^2^	0.0118	1	0.0118	87.86	<0.0001	
B^2^	0.0045	1	0.0045	33.78	<0.0001	
C^2^	0.0099	1	0.0099	73.56	<0.0001	
D^2^	0.0080	1	0.0080	59.36	<0.0001	
Residual	0.0016	12	0.0001			
Lack of Fit	0.0015	10	0.0001	2.66	0.3039	not significant
Pure Error	0.0001	2	0.0001			
Cor Total	0.0441	26				

R^2^ = 0.9635; Predicted R^2^ = 0.7985; C.V.% = 1.22; Adjusted R^2^ = 0.9208; Adeq Precision = 14.7255.

**Table 12:** Analysis of variance (ANOVA) for the quadratic model in the Box–Behnken design (RC).

Source	Sum of Squares	df	Mean Square	F-Value	*p*-Value	
Model	0.0070	10	0.0007	23.44	<0.0001	significant
A-Pc	0.0019	1	0.0019	63.57	<0.0001	
B-Pp	0.0016	1	0.0016	55.26	<0.0001	
C-Vy	0.0016	1	0.0016	53.01	<0.0001	
D-f	2.341 × 10^−6^	1	2.341 × 10^−6^	0.0785	0.7829	
AB	0.0004	1	0.0004	14.03	0.0018	
AC	0.0004	1	0.0004	14.79	0.0014	
AD	4.622 × 10^−6^	1	4.622 × 10^−6^	0.1551	0.6989	
BC	0.0000	1	0.0000	0.7729	0.3923	
BD	0.0007	1	0.0007	24.00	0.0002	
CD	0.0003	1	0.0003	8.75	0.0093	
Residual	0.0005	16	0.0000			
Lack of Fit	0.0005	14	0.0000	3.77	0.2293	not significant
Pure Error	0.0000	2	8.71 × 10^−6^			
Cor Total	0.0075	26				

R^2^ = 0.9361; Predicted R^2^ = 0.8036; C.V.% = 8.08; Adjusted R^2^ = 0.8962; Adeq Precision = 19.8140.

**Table 13:** Optimization goals for the effectiveness of hybrid laser cleaning of carbon deposits.

Name	Goal	Limits	Weights
CW laser power Pc/%	In range	15–25	1
Pulsed laser power Pp/%	In range	75–85	1
Y_Scanning speed/mm·s^−1^	In range	15–25	1
Repetition frequency f/kHz	In range	30–40	1
Carbon residue rate RC	Minimize	0–0.05	1
Surface roughness Sa/μm	Minimize	0.4–1	1

**Table 14:** Error analysis of validation test results.

Sample	Surface Roughness/Sa (μm)			Carbon Residue Rate/RC		
	Optimize Values	Experimental Values	Relative Errors	Optimize Values	Experimental Values	Relative Errors
1	0.947	0.970	2.37%	3.65%	3.87%	5.68%
2	0.947	0.935	1.28%	3.65%	3.58%	1.96%
3	0.947	0.959	1.25%	3.65%	3.47%	5.19%
Prediction Range	0.915–0.978			0.0225–0.0509		

## Data Availability

The original contributions presented in this study are included in the article, and further inquiries can be directed to the corresponding author.
